# A Study of Pro-Bnp Compared to Procalcitonin in Patient With Severe Sepsis As a Marker of Diagnosis of Sepsis in Critically Ill Patients

**DOI:** 10.1186/2197-425X-3-S1-A515

**Published:** 2015-10-01

**Authors:** SR Elshrief, AM Fayed, AAEA Mahros, AAEG Aglan

**Affiliations:** Alexandria University, Faculty of Medicine, Critical Care Medicine, Alexandria, Egypt

## Introduction

Early diagnosis of sepsis is the key for improving the survival, therefore biomarkers have an important place in this process because they can indicate the presence or absence of sepsis [1]. Procalcitonin aids in diagnosis and risk stratification of bacterial sepsis [2].Using pro-BNP as inflammatory marker in diagnosing severe sepsis and septic shock is based on cytokines production independent of myocardial dysfunction. Bacterial endotoxins secreted from Gram-negative organisms and *Staphylococcus aureus* alpha toxin increase the BNP messenger RNA expression. Also the activity of Neutral endopeptidase 24.11 the enzyme responsible for degradation of proBNP decreases in septic shock. Finally, proBNP clearance decreases due to renal dysfunction [3].

## Objectives

In this study we compared the diagnostic value of ProBNP and procalcitonin in severe sepsis patients.

## Methods

24 patients with the preliminary diagnosis of severe sepsis were divided into two groups according to Echocardiography (Echo) findings, cultures from suspected sites of infection were done. Blood levels of CRP, ProBNP and PCT were measured on admission. We compared ProBNP and PCT levels in severe sepsis patients with normal Echo findings and those with systolic and/or diastolic dysfunction and we also compared level of ProBNP in these two groups with the control group.

## Results

Level of proBNP was very high in total patients compared to control group. Level of proBNP to diagnose severe sepsis in patients group (with cutoff point >108pg/ml as recommended by Youden’s index) had 95.83% sensitivity, 83.33% specificity with 92.0% PPV, 90.91% NPV and 91.67% accuracy.To differentiate severe sepsis patients with normal Echo findings from those with abnormal Echo findings, ProBNP (with cutoff point >2900 pg/ml) had 41.67% sensitivity, 83.33% specificity, 71.43% PPV, and 58.82% NPV with 62.50% accuracy and PCT (with cutoff point >2 pg/ml) had 66.67% sensitivity, 50.0% specificity, 57.14% PPV, 60% NPV with 58.33% accuracy.

## Conclusions

ProBNP level increased in all patients with severe sepsis and septic shock with or without cardiac dysfunction, so ProBNP might be a good marker for severe sepsis. ProBNP level was a good marker for systolic dysfunction but ProBNP or PCT levels could not differentiate between severe sepsis patients with or without cardiac dysfunction.

## Grant Acknowledgment

Special thanks to Dr Akram Fayed.
